# Recognition of aerosol transmission of infectious agents: a commentary

**DOI:** 10.1186/s12879-019-3707-y

**Published:** 2019-01-31

**Authors:** Raymond Tellier, Yuguo Li, Benjamin J. Cowling, Julian W. Tang

**Affiliations:** 1 Department of Pathology and Laboratory Medicine, University of Calgary, Calgary, AB Canada; 2 Department of Mechanical Engineering, University of Hong Kong, Pokfulam, Hong Kong, Special Administrative Region of China; 3 WHO Collaborating Centre for Infectious Disease Epidemiology and Control, School of Public Health, The University of Hong Kong, Pokfulam, Hong Kong, Special Administrative Region of China; 4 Department of Infection, Immunity and Inflammation, University of Leicester, Leicester, UK; 5 Clinical Microbiology, University Hospitals of Leicester NHS Trust, Level 5 Sandringham Building, Leicester Royal Infirmary, Infirmary Square, Leicester, LE1 5WW UK

**Keywords:** Aerosol, Airborne, Droplet, Transmission, Infection

## Abstract

Although short-range large-droplet transmission is possible for most respiratory infectious agents, deciding on whether the same agent is also airborne has a potentially huge impact on the types (and costs) of infection control interventions that are required.

The concept and definition of aerosols is also discussed, as is the concept of large droplet transmission, and airborne transmission which is meant by most authors to be synonymous with aerosol transmission, although some use the term to mean either large droplet or aerosol transmission.

However, these terms are often used confusingly when discussing specific infection control interventions for individual pathogens that are accepted to be mostly transmitted by the airborne (aerosol) route (e.g. tuberculosis, measles and chickenpox). It is therefore important to clarify such terminology, where a particular intervention, like the type of personal protective equipment (PPE) to be used, is deemed adequate to intervene for this potential mode of transmission, i.e. at an N95 rather than surgical mask level requirement.

With this in mind, this review considers the commonly used term of ‘aerosol transmission’ in the context of some infectious agents that are well-recognized to be transmissible via the airborne route. It also discusses other agents, like influenza virus, where the potential for airborne transmission is much more dependent on various host, viral and environmental factors, and where its potential for aerosol transmission may be underestimated.

## Background

The classification of an infectious agent as airborne and therefore ‘aerosol-transmissible’ has significant implications for how healthcare workers (HCWs) need to manage patients infected with such agents and what sort of personal protective equipment (PPE) they will need to wear. Such PPE is usually more costly for airborne agents (i.e. aerosol-transmissible) than for those that are only transmitted by large droplets or direct contact because of two key properties of aerosols: a) their propensity to follow air flows, which requires a tight seal of the PPE around the airways, and b) for bioaerosols, their small size, which calls for an enhanced filtering capacity.

Several recent articles and/or guidance, based on clinical and epidemiological data, have highlighted the potential for aerosol transmission for Middle-East Respiratory Syndrome-associated coronavirus (MERS-CoV) [1, 2] and Ebola virus [3, 4]. Some responses to the latter have attempted to put these theoretical risks in a more practical light [4], and this nicely illustrates the quandary of how to classify such emerging or re-emerging pathogens into either the large droplet (short-range) versus airborne (short and possibly long-range) transmission categories. However, this delineation is not black and white, as there is also the potential for pathogens under both classifications to be potentially transmitted by aerosols between people at close range (i.e. within 1 m).

### Definitions

Strictly speaking, ‘aerosols’ refer to particles in suspension in a gas, such as small droplets in air. There have been numerous publications classifying droplets using particle sizes over the years [5–10]. For example it is generally accepted that: i) small particles of < 5–10 μm aerodynamic diameter that follow airflow streamlines are potentially capable of short and long range transmission; particles of < 5 μm readily penetrates the airways all the way down to the alveolar space, and particles of < 10 μm readily penetrates below the glottis (7) ii) large droplets of diameters > 20 μm refer to those that follow a more ballistic trajectory (i.e. falling mostly under the influence of gravity), where the droplets are too large to follow inhalation airflow streamlines. For these particle sizes, for example, surgical masks would be effective, as they will act as a direct physical barrier to droplets of this size that are too large to be inhaled into the respiratory tract around the sides of the mask (which are not close-fitting); iii) ‘intermediate particles’ of diameters 10–20 μm, will share some properties of both small and large droplets, to some extent, but settle more quickly than particles < 10 μm and potentially carry a smaller infectious dose than large (> 20 μm) droplets.

‘Aerosols’ would also include ‘droplet nuclei’ which are small particles with an aerodynamic diameter of 10 μm or less, typically produced through the process of rapid desiccation of exhaled respiratory droplets [5, 6]. However, in some situations, such as where there are strong ambient air cross-flows, for example, larger droplets can behave like aerosols with the potential to transmit infection via this route (see next section below).

Several properties can be inferred from this, for example the penetration of the lower respiratory tract (LRT), as at greater than 10 μm diameter, penetration below the glottis rapidly diminishes, as does any potential for initiating an infection at that site. Similarly, any such potential for depositing and initiating an LRT infection is less likely above a droplet diameter of 20 μm, as such large particles will probably impact onto respiratory epithelial mucosal surfaces or be trapped by cilia before reaching the LRT [6].

The Infectious Diseases Society of America (IDSA) has proposed a scheme that is essentially equivalent [7], defining “respirable particles” as having a diameter of 10 μm or less; and “inspirable particles” as having a diameter between 10 μm and 100 μm, nearly all of which are deposited in the upper airways. Some authors have proposed the term “fine aerosols”, consisting of particles of 5 μm or less, but this has been in part dictated by constraints from measurement instruments [8]. Several authors lump together transmission by either large droplets or aerosol-sized particles as “airborne transmission” [9], or use “aerosol transmission” to describe pathogens that can cause disease via inspirable particles of any size [10].

However, we think that it is important to maintain a distinction between particles of < 10 μm and larger particles, because of their significant qualitative differences including suspension time, penetration of different regions of the airways and requirements for different PPE. In this commentary, we use the common convention of “airborne transmission” to mean transmission by aerosol-size particles of < 10 μm.

**Fig. 1:**
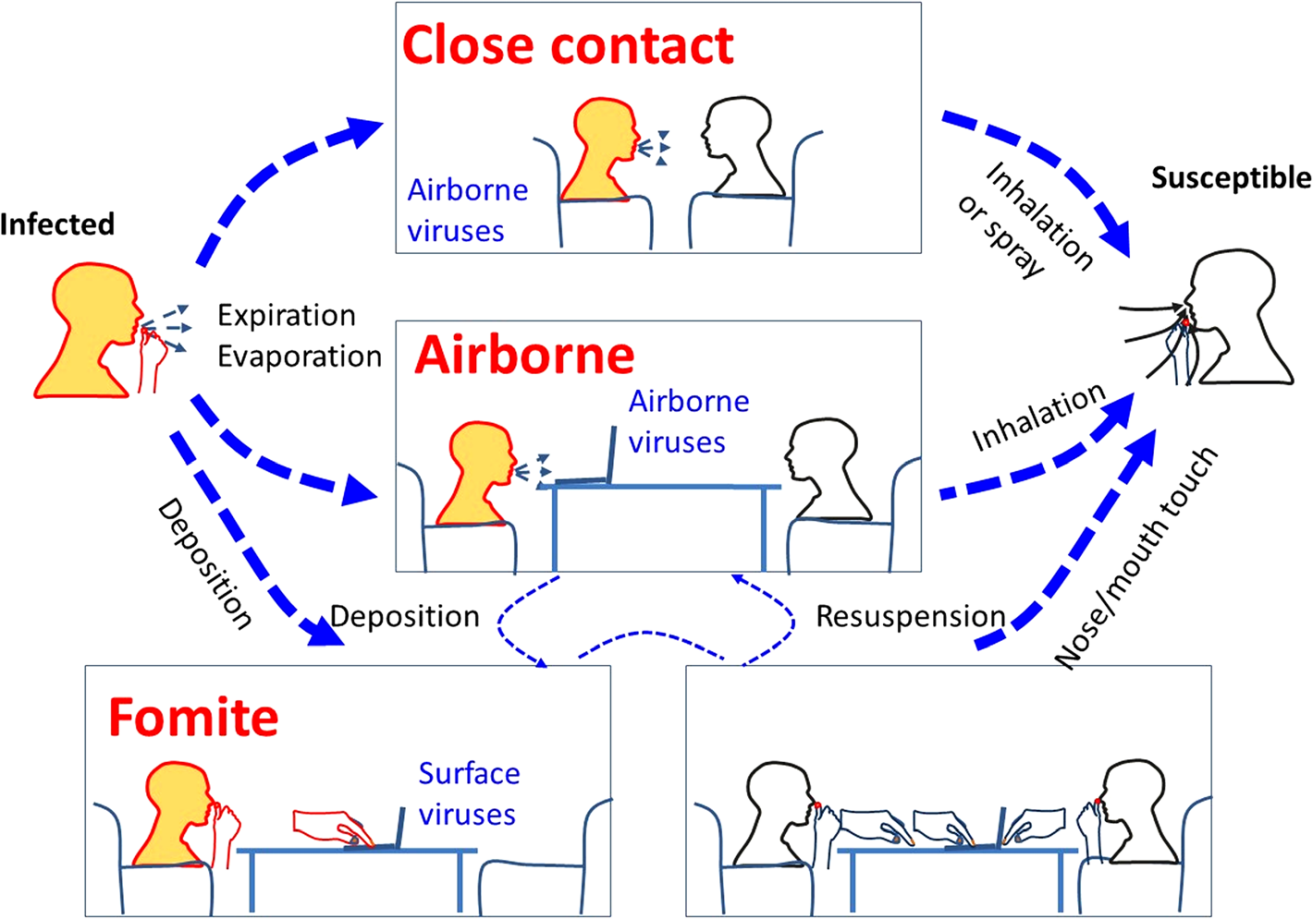
An illustration of various possible transmission routes of respiratory infection between an infected and a susceptible individual. Both close range (i.e. conversational) airborne transmission and longer range (over several meters) transmission routes are illustrated here. The orange head colour represents a source and the white head colour a potential recipient (with the bottom right panel indicating that both heads are potential recipients via self-inoculation from contaminated surface fomite sources). Here ‘Expiration’ also includes normal breathing exhalation, as well as coughing and/or sneezing airflows. Airborne droplets can then settle on surfaces (fomites) from where they can be touched and carried on hands leading to further self-inoculation routes of transmission

If the infected patients produce infectious droplets of varying sizes by breathing, coughing or sneezing, transmission between individuals by both short-range large droplets and airborne small droplet nuclei are both possible, depending on the distance from the patient source. Figure 1 illustrates these potential routes of short and long-range airborne transmission, as well as the downstream settling of such droplets onto surfaces (fomites). From such fomites, they may be touched and transported by hands to be self-inoculated into mucosal membranes e.g. in the eyes, nose and mouth) to cause infection, depending on the survival characteristics of individual pathogens on such surfaces, and the susceptibility (related to available, compatible cell receptors) of the different exposed tissues to infection by these pathogens.

For example, when the infectious dose (the number of infectious agents required to cause disease) of an organism is low, and where large numbers of pathogen-laden droplets are produced in crowded conditions with poor ventilation (in hospital waiting rooms, in lecture theatres, on public transport, etc.), explosive outbreaks can still occur, even with pathogens whose airborne transmission capacity is controversial, e.g. the spread of influenza in a grounded plane where multiple secondary cases were observed in the absence of any ventilation [11].

The more mechanistic approaches (i.e. arguing from the more fundamental physical and dynamic behavior of small versus larger particle and droplet sizes in the absence of any biological interactions) to classifying which pathogens are likely to transmit via the airborne route have been published in various ways over the years [12–17], but may have to be considered in combination with epidemiological and environmental data to make a convincing argument about the potential for the airborne transmissibility of any particular agent – and the number of possible potential exposure scenarios is virtually unlimited).

### The importance of ambient airflows and the of aerosols

One should note that “aerosol” is essentially a relative and not an absolute term. A larger droplet can remain airborne for longer if ambient airflows can sustain this suspension for longer, e.g. in some strong cross-flow or natural ventilation environments, where ventilation-induced airflows can propagate suspended pathogens effectively enough to cause infection at a considerable distance away from the source.

One of the standard rules (Stoke’s Law) applied in engineering calculations to estimate the suspension times of droplets falling under gravity with air resistance, was derived assuming several conditions including that the ambient air is still [13–17]. So actual suspension times will be far higher where there are significant cross-flows, which is often the case in healthcare environments, e.g. with doors opening, bed and equipment movement, and people walking back and forth, constantly. Conversely, suspension times, even for smaller droplet nuclei, can be greatly reduced if they encounter a significant downdraft (e.g. if they pass under a ceiling supply vent). In addition, the degree of airway penetration, for different particle sizes, also depends on the flow rate.

In the field of dentistry and orthopedics, where high-powered electric tools are used, even bloodborne viruses (such as human immunodeficiency virus – HIV, hepatitis B and hepatitis B viruses) can become airborne when they are contained in high velocity blood splatter generated by these instruments [18, 19]. Yet, whether they can cause efficient transmission via this route is more debatable. This illustrates another point, that although some pathogens can be airborne in certain situations, they may not necessarily transmit infection and cause disease via this route.

### Outline

Over time, for a pathogen with a truly predominant airborne transmission route, eventually sufficient numbers of published studies will demonstrate its true nature [13]. If there are ongoing contradictory findings in multiple studies (as with influenza virus), it may be more likely that the various transmission routes (direct/indirect contact, short-range droplet, long-, and even short-range airborne droplet nuclei) may predominate in different settings [16, 20], making the airborne route for that particular pathogen more of an opportunistic pathway, rather than the norm [21]. Several examples may make this clearer.

The selected pathogens and supporting literature summarized below are for illustrative purposes only, to demonstrate how specific studies have impacted the way we consider such infectious agents as potentially airborne and ‘aerosol-transmissible’. It is not intended to be a systematic review, but rather to show how our thinking may change with additional studies on each pathogen, and how the acceptance of “aerosol transmission” for different pathogens did not always followed a consistent approach.

## Results and discussion

### Chickenpox

Chickenpox is a febrile, vesicular rash illness caused by varicella zoster virus (VZV), a lipid-enveloped, double-stranded DNA virus, and a member of the *Herpesviridae* family.

For chickenpox, the evidence appears to be mainly epidemiological and clinical, though this has appeared to be sufficient to classify varicella zoster virus (VZV) as an airborne agent. Studies on VZV have shown that the virus is clearly able to travel long distances (i.e. up to tens of meters away from the index case, to spread between isolation rooms and other ward areas connected by corridors, or within a household) to cause secondary infections and/or settle elsewhere in the environment [22–24]. In addition, Tang et al. [25] showed that airborne VZV could leak out of isolation rooms transported by induced environmental airflows to infect a susceptible HCW, most likely via the direct inhalation route.

### Measles

Measles (also known as rubeola) is a febrile, rash illness caused by the measles virus, a lipid-enveloped, single-stranded, negative-sense RNA virus, and a member of the *Paramyxoviridae* family.

For measles several studies examined a more mechanistic airflow dynamical explanation (i.e. based upon the fundamental physics and behaviour of airborne particles) for the main transmission route involved in several measles outbreaks [26], including that of Riley and colleagues who used the concept of ‘quanta’ of infection [27]. Later, two other outbreaks in outpatient clinics included retrospective airflow dynamics analysis, providing more evidence for the transmissibility of measles via the airborne route [28, 29].

### Tuberculosis

Tuberculosis is a localized or systemic, but most often respiratory bacterial illness caused by mycobacteria belonging to the *Mycobacterium tuberculosis* complex.

For tuberculosis (TB), definitive experimental evidence of airborne transmission being necessary and sufficient to cause disease was provided in a series of guinea-pig experiments [30, 31], which has been repeated more recently in a slightly different clinical context [32]. Numerous other outbreak reports have confirmed the transmissibility of TB via the airborne route [33–35], and interventions specifically targeting the airborne transmission route have proven effective in reducing TB transmission [36].

### Smallpox

Smallpox is a now eradicated, febrile, vesicular rash and disseminated illness, caused by a complex, double-stranded DNA orthopoxvirus (*Poxviridae* family), which can present clinically in two forms, as variola major or variola minor.

For smallpox, a recent comprehensive, retrospective analysis of the literature by Milton has suggested an important contribution of the airborne transmission route for this infection [37]. Although various air-sampling and animal transmission studies were also reviewed, Milton also emphasized clinical epidemiological studies where non-airborne transmission routes alone could not account for all the observed smallpox cases.

At least one well-documented hospital outbreak, involving 17 cases of smallpox, could only be explained by assuming the aerosol spread of the virus from the index case, over several floors. Retrospective smoke tracer experiments further demonstrated that airborne virus could easily spread to patients on different floors via open windows and connecting corridors and stairwells in a pattern roughly replicating the location of cases [38].

### Emerging coronaviruses: Severe acute respiratory syndrome (SARS), middle-east respiratory syndrome (MERS)

Coronaviruses are lipid-enveloped, single-stranded positive sense RNA viruses, belong to the genus *Coronavirus* and include several relatively benign, seasonal, common cold viruses (229E, OC43, NL63, HKU-1). They also include two new more virulent coronaviruses: severe acute respiratory syndrome coronavirus (SARS-CoV), which emerged in the human population in 2003; and Middle-East Respiratory Syndrome coronavirus (MERS-CoV), which emerged in humans during 2012.

For SARS-CoV, several thorough epidemiological studies that include retrospective airflow tracer investigations are consistent with the hypothesis of an airborne transmission route [39–41]. Air-sampling studies have also demonstrated the presence of SARS-CoV nucleic acid (RNA) in air, though they did not test viability using viral culture [42].

Although several studies compared and contrasted SARS and MERS from clinical and epidemiological angles [43–45], the predominant transmission mode was not discussed in detail, if at all. Several other studies do mention the potential for airborne transmission, when comparing potential routes of infection, but mainly in relation to super-spreading events or “aerosolizing procedures”such as broncho-alveolar lavage, and/or a potential route to take into consideration for precautionary infection control measures [46–48]. However, from the various published studies, for both MERS and SARS, it is arguable that a proportion of transmission occurs through the airborne route, although this may vary in different situations (e.g. depending on host, and environmental factors). The contribution from asymptomatic cases is also uncertain [49].

For both SARS and MERS, LRT samples offer the best diagnostic yield, often in the absence of any detectable virus in upper respiratory tract (URT) samples [50–52]. Furthermore, infected, symptomatic patients tend to develop severe LRT infections rather than URT disease. Both of these aspects indicate that this is an airborne agent that has to penetrate directly into the LRT to preferentially replicate there before causing disease.

For MERS-CoV specifically, a recent study demonstrated the absence of expression of dipeptidyl peptidase 4 (DPP4), the identified receptor used by the virus, in the cells of the human URT. The search for an alternate receptor was negative [53]. Thus, the human URT would seem little or non-permissive for MERS-CoV replication, indicating that successful infection can only result from the penetration into the LRT via direct inhalation of appropriately sized ‘droplet nuclei’-like’ particles. This makes any MERS-CoV transmission leading to MERS disease conditional on the presence of virus-containing droplets small enough to be inhaled into the LRT where the virus can replicate.

### Influenza

Influenza is a seasonal, often febrile respiratory illness, caused by several species of influenza viruses. These are lipid-enveloped, single-stranded, negative-sense, segmented RNA viruses belonging to the *Orthomyxoviridae* family. Currently, influenza is the only common seasonal respiratory virus for which licensed antiviral drugs and vaccines are available.

For human influenza viruses, the question of airborne versus large droplet transmission is perhaps most controversial [54–57]. In experimental inoculation experiments on human volunteers, aerosolized influenza viruses are infectious at a dose much lower than by nasal instillation [58]. The likely answer is that both routes are possible and that the importance and significance of each route will vary in different situations [16, 20, 21].

For example, tighter control of the environment may reduce or prevent airborne transmission by: 1) isolating infectious patients in a single-bed, negative pressure isolation room [25]; 2) controlling environmental relative humidity to reduce airborne influenza survival [59]; 3) reducing exposure from aerosols produced by patients through coughing, sneezing or breathing with the use of personal protective equipment (wearing a mask) on the patient (to reduce source emission) and/or the healthcare worker (to reduce recipient exposure) [60]; 4) carefully controlling the use and exposure to any respiratory assist devices (high-flow oxygen masks, nebulizers) by only allowing their use in designated, containment areas or rooms [61]. The airflows being expelled from the side vents of oxygen masks and nebulisers will contain a mixture of patient exhaled air (which could be carrying airborne pathogens) and incoming high flow oxygen or air carrying nebulized drugs. These vented airflows could then act as potential sources of airborne pathogens.

Numerous studies have shown the emission of influenza RNA from the exhaled breath of naturally influenza-infected human subjects [62–66] and have detected influenza RNA in environmental air [67–69]. More recently, some of these studies have shown the absence of [70], or significantly reduced numbers of viable viruses in air-samples with high influenza RNA levels (as tested by PCR) [66, 71, 72]. The low number of infectious particles detected is currently difficult to interpret as culture methods are inherently less sensitive than molecular methods such as PCR, and the actual operation of air-sampling itself, through shear-stress related damage to the virions, also causes a drop in infectivity in the collected samples. This may lead to underestimates of the amount of live virus in these environmental aerosols.

An additional variable to consider is that some animal studies have reported that different strains of influenza virus may vary widely in their capacity for aerosol transmission [73].

In some earlier articles that discuss the predominant mode of influenza virus transmission [74–78], these same questions are addressed with mixed conclusions. Most of the evidence described to support their views was more clinical and epidemiological, and included some animal and human volunteer studies, rather than physical and mechanistic. Yet, this mixed picture of transmission in different circumstances is probably the most realistic.

It is noteworthy that several infections currently accepted as airborne-transmitted, such as measles, chickenpox or TB present, in their classical form, an unmistakable and pathognomonic clinical picture. In contrast the clinical picture of influenza virus infection has a large overlap with that of other respiratory viruses, and mixed outbreaks have been documented [79]. Thus, a prevalent misconception in the field has been to study ‘respiratory viruses’ as a group. However, given that these viruses belong to different genera and families, have different chemical and physical properties and differing viral characteristics, it is unwise and inaccurate to assume that any conclusions about one virus can be applied to another, e.g. in a Cochrane review of 59 published studies on interventions to reduce the spread of respiratory viruses, there were actually only two studies specifically about influenza viruses [80]. As the authors themselves pointed out, no conclusion specific to influenza viruses was possible.

While many airborne infections are highly contagious, this is not, strictly speaking, part of the definition. Even so, the lower contagiousness of influenza compared to, say, measles has been invoked as an argument against a significant contribution of airborne transmission. Yet, it should be noted that a feature of influenza virus infections is that the incubation time (typically 1–2 days) is much shorter than its duration of shedding. This allows for the possibility that a susceptible person will be exposed during an outbreak to several different infectious cases belonging to more than one generation in the outbreak. This multiple exposure and telescoping of generations may result in an underestimate of influenza virus transmissibility, as fewer secondary cases will be assigned to a known index case, when in fact the number of secondary cases per index could be much higher. For example, it is known that in some settings a single index case can infect a large number of people, e.g. 38 in an outbreak on an Alaska Airlines flight [11].

### Ebola

Ebola is a viral hemorrhagic fever associated with a very high mortality, caused by the Ebola viruses; these are enveloped single-strand, negative-sense RNA viruses comprising five species within the family *Filoviridae*. Four Ebola species have been implicated in human diseases; the most widespread outbreak, also the most recent, was caused by Ebola Zaire in West Africa in 2013–2016. The transmission of Ebola viruses has been reviewed in depth by Osterholm et al. (4). These authors noted the broad tissue tropism, as well as the high viral load reached during illness and the low infectious dose, from which it appears inescapable that more than one mode of transmission is possible.

Regarding aerosol transmission, concerns are raised by several documented instances of transmission of Ebola Zaire in laboratory settings between animals without direct contact [81, 82] (also reviewed in [4]). Experimental infections of Rhesus monkeys by Ebola Zaire using aerosol infection has been shown to be highly effective [83, 84] and this experimental procedure has in fact been used as infectious challenge in Ebola vaccine studies [85, 86]. Rhesus monkeys infected by aerosol exposure reliably developed disseminated, fatal infection essentially similar to that caused by parenteral infection with the addition of involvement of the respiratory tract. Autopsies showed pathological findings in the respiratory tract and respiratory lymphoid system in animals infected by the aerosol route that are not found in animals infected parenterally [83, 84].

Such respiratory pathological lesions have not been reported in human autopsies of Ebola cases, but as noted by Osterholm et al. [4], there have been few human autopsies of Ebola cases, arguably too few to confidently rule out any possibility of disease acquired by the aerosol route. The precautionary principle would therefore dictate that aerosol precautions be used for the care of infected patients, and especially considering that infection of the respiratory tract in such patients is not necessary to create an aerosol hazard: Ebola viruses reach a very high titer in blood or other bodily fluids during the illness [87, 88] and aerosolization of blood or other fluids would create a significant airborne transmission hazard.

## Conclusions

In summary, despite the various mechanistic arguments about which organisms can be potentially airborne and therefore aerosol-transmissible, ultimately, the main deciding factor appears to be how many studies using various differing approaches: empirical (clinical, epidemiological), and/or experimental (e.g. using animal models), and/or mechanistic (using airflow tracers and air-sampling) methods, reach the same consensus opinion. Over time, the scientific community will eventually form an impression of the predominant transmission route for that specific agent, even if the conclusion is one of mixed transmission routes, with different routes predominating depending on the specific situations. This is the case for influenza viruses, and is likely the most realistic.

Some bacterial and viral infections that have more than one mode of transmission are also *anisotropic*, like anthrax, plague, tularemia and smallpox: the severity of the disease varies depending on the mode of transmission [37, 89]. Older experimental infection experiments on volunteers suggest that this is the case for influenza, with transmission by aerosols being associated with a more severe illness [14, 90], and some more recent field observations are consistent with this concept [57]. For anisotropic agents, even if a mode of transmission (e.g. aerosols) accounts for only a minority of cases, interruption of that route of transmission may be required if it accounts for the most severe cases.

## Abbreviations

LRT: lower respiratory tract
MERS-CoV: Middle East Respiratory Syndrome-associated coronavirus
PCR: polymerase chain reaction
RNA: ribonucleic acid
SARS-CoV: severe acute respiratory syndrome-associated coronavirus
TB: tuberculosis
URT: upper respiratory tract
VZV: varicella zoster virus
